# HupZ, a Unique Heme-Binding Protein, Enhances Group A Streptococcus Fitness During Mucosal Colonization

**DOI:** 10.3389/fcimb.2022.867963

**Published:** 2022-06-14

**Authors:** Kristin V. Lyles, Lamar S. Thomas, Corbett Ouellette, Laura C. C. Cook, Zehava Eichenbaum

**Affiliations:** ^1^ Department of Biology, Georgia State University, Atlanta, GA, United States; ^2^ Binghamton Biofilm Research Center, Department of Biology, Binghamton University, Binghamton, NY, United States

**Keywords:** Group A Streptococcus, HupZ, heme, heme utilization, heme toxicity, iron, mice colonization

## Abstract

Group A Streptococcus (GAS) is a major pathogen that causes simple and invasive infections. GAS requires iron for metabolic processes and pathogenesis, and heme is its preferred iron source. We previously described the iron-regulated *hupZ* in GAS, showing that a recombinant HupZ-His_6_ protein binds and degrades heme. The His_6_ tag was later implicated in heme iron coordination by HupZ-His_6_. Hence, we tested several recombinant HupZ proteins, including a tag-free protein, for heme binding and degradation *in vitro*. We established that HupZ binds heme but without coordinating the heme iron. Heme-HupZ readily accepted exogenous imidazole as its axial heme ligand, prompting degradation. Furthermore, HupZ bound a fragment of heme c (whose iron is coordinated by the cytochrome histidine residue) and exhibited limited degradation. GAS, however, did not grow on a heme c fragment as an iron source. Heterologous HupZ expression in *Lactococcus lactis* increased heme b iron use. A GAS *hupZ* mutant showed reduced growth when using hemoglobin as an iron source, increased sensitivity to heme toxicity, and decreased fitness in a murine model for vaginal colonization. Together, the data demonstrate that HupZ contributes to heme metabolism and host survival, likely as a heme chaperone. HupZ is structurally similar to the recently described heme c-degrading enzyme, Pden_1323, suggesting that the GAS HupZ might be divergent to play a new role in heme metabolism.

## Introduction

Group A Streptococcus (GAS, or *Streptococcus pyogenes)* is an obligate human pathogen that primarily infects the skin and the upper respiratory system. GAS can also produce invasive, systemic diseases, including Streptococcal toxic shock syndrome and necrotizing fasciitis, both with high mortality rates (Walker et al., 2014). In some cases, superficial GAS infections can cause harmful immune responses leading to post-streptococcal sequelae like glomerulonephritis and rheumatic heart disease (Watkins et al., 2017). There was a marked increase in invasive GAS infections in the United States and Europe in the 1980s with the emergence of more virulent strains, particularly the M1T1 strain (Barnett et al., 2018). The rise in invasive infections is of particular concern since there is no vaccine, and GAS is becoming increasingly resistant to tetracycline and macrolides (Davies et al., 2015; CDC, 2019).

There is very little free iron in the human body; much of the metal is sequestered by proteins that facilitate transport and storage and reduce iron-mediated toxicity (Marchetti et al., 2020). Most iron in the body is bound to a porphyrin ring, called heme, and two-thirds of the body heme is found in hemoglobin (i.e., heme b). There are other types of heme in the body; for example, heme c is bound to cytochrome c and differs from heme b in that it is covalently bound to a proteinaceous region. For clarity, in this manuscript, we will use “heme” to refer to heme b.

Iron-requiring pathogens, such as GAS, have evolved mechanisms to obtain heme iron from the host. GAS hemolysins lyse erythrocytes and other cell types, releasing heme and hemoproteins, such as hemoglobin and cytochromes. The *sia* and *hupYZ* operons allow GAS to acquire heme from various hemoproteins and transport it into the cell (Bates et al., 2003; Sun et al., 2010; Chatterjee et al., 2020). The metalloregulator, MtsR, controls both the *sia* and *hupYZ* operons, permitting elevated expression in low-iron conditions (Bates et al., 2005). This regulon is also upregulated during vaginal colonization of GAS in mice (Cook et al., 2019). How heme is degraded by GAS is not known, but the putative cytoplasmic protein HupZ was implicated in the process (Sachla et al., 2016).

Many organisms use heme oxygenases to degrade heme and release the iron. The first heme oxygenase (HO-1) was identified in mammals (Tenhunen et al., 1969). Subsequently, homologs were identified in several bacterial species, such as HmuO of *Corynebacterium diptheriae*, HemO of *Neisseria menigitidis*, and HemO/PigA of *Pseudomonas aeruginosa* (Schmitt, 1997; Zhu et al., 2000; Ratliff et al., 2001). These enzymes degrade heme through the canonical or HO-1-like pathway, which consists of three oxygenation steps resulting in equal amounts of α-biliverdin, ferrous iron, and carbon monoxide (CO) (Wilks and Heinzl, 2014; Wilks and Ikeda-Saito, 2014; Lyles and Eichenbaum, 2018). The first noncanonical heme oxygenases, IsdG, and its homolog IsdI were identified in *Staphylococcus aureus* (Skaar et al., 2004). The IsdG/I reaction yields a mixture of β- and δ-staphylobilin and releases formaldehyde instead of CO. Some pathogenic bacteria utilize proteins from the flavin mononucleotide (FMN)-binding subfamily for heme-binding or degradation, such as HugZ from *Helicobacter pylori* (which produces δ-biliverdin) and ChuZ from *Campylobacter jejuni* (Hu et al., 2011; Zhang et al., 2011). Pden_1323, from *Paracoccus denitrificans*, is a member of this family, and while it lacks the conserved axial heme ligand from HugZ and ChuZ, it can degrade fragments of heme c (Li et al., 2021).

GAS HupZ shares structural similarity to the HugZ family (Sachla et al., 2016). HupZ purified with a His_6_-tag binds and degraded heme *in vivo*, releasing CO, free iron, and an unidentified chromophore. Further investigation of the recombinant protein using EPR and resonance Raman spectroscopy indicated that a histidine residue coordinated the heme iron, yet site mutation of the only histidine residue in HupZ did not affect the spectra (Traore et al., 2021). These observations suggested that the His_6_-tag facilitated the heme-binding and degradation exhibited by the HupZ-His_6_ protein. Here, we investigate heme-binding and degradation by HupZ expressed without a His_6_ tag and use mutagenesis, heterologous expression, and a mice model to probe the protein’s function *in vivo*. The data confirm that HupZ plays a role in heme use and tolerance in GAS and suggest that HupZ is a member of an emerging group of heme-binding proteins in bacteria.

## Materials and Methods

### Strains, Media, and Chemicals

*E. coli* were grown at 37°C aerobically (225 rpm) in Luria-Bertani (LB) broth or agar, supplemented with appropriate antibiotics. GAS was grown statically in Todd Hewitt yeast broth (THYB, 5 ml of media in 15-ml screw-top tubes, Thermo Scientific #33965) or agar (THYA) at 37°C. *L. lactis* was grown statically in GM17 at 30°C (10 ml of media in 15-ml screw-top tubes). Plasmid extractions were performed using the Promega Wizard Miniprep kit (PR-A7510) or the Qiagen Midiprep kit (12123). Genomic DNA was harvested with Invitrogen PureLink (K1820-01). Unless otherwise specified, chemicals were purchased from Sigma.

### Plasmid Construction

A list of strains and plasmids can be found in **Table 1**, and primer sequences are shown in **Table 2**. Plasmid engineering was confirmed with restriction digest and PCR analyses.

**Table 1:** Strains and plasmids.

Strains	Relevant properties	Source/reference
M49Rescue	NZ131 wild-type rescue strain	This study
M49Lyles	NZ131 containing *hupZ::cm^R^* mutation	This study
M49Lyles + pKV127	NZ131 containing *hupZ::cm^R^* mutation and pKV127	This study
pDC123	Source of Cm^R^ allele	(Chaffin and Rubens, 1998)
pET/His_6_/MBP/TEV	N-terminal His_6_ followed by MBP, P_T7_, Kan^R^	Addgene plasmid #29656
pET MBP TEV	N-terminal MBP, P_T7_, Amp^R^	Addgene plasmid #48311
pJRS700	pVE6037 derivative, Kan^R^, TM^S^	(Bates et al., 2005)
pKV102	Expresses HupZ-Strep from P_T7_	Vector Builder
pKV105	pNZ8008 derivative expresses *hupZ*, P^nisA^, CM^R^	This study
pKV111	pUC19 derivative containing *hupZ::cm^R^* allele, Amp^R^	This study
pKV113	pKV111 derivative with a site mutation to add *Eco*RI site downstream of *hupZ::cm^R^* allele	This study
pKV117	pJRS700 derivative containing *hupZ::cm^R^* allele, Kan^R^, TM^S^	This Study
pKV135	pET MBP TEV derivative that expresses MBP-HupZ from P_T7_	This Study
pKV138	pLC007 derivative expressing *hupZ*, Spec^R^, P_RecA_	This study
pKV141	pLC007 derivative *hupY* deleted, Spec^R^, P_RecA_	This study
pLC007	Expresses *hupY*, Spec^R^, P_RecA_	(Cook et al., 2019)
pNZ8008	pSH71 replicon with promoterless *gusA* gene, P_nisA_, Cm^r^	(de Ruyter et al., 1996)
pNZ9530	pAMb1 replicon expressing *nisR* and *nisK*, Ery_R_, P_RepA_	(Kleerebezem et al., 1997)
pRK793	p15A replicon expressing SuperTev, P_tac_, Kan^R^, CM^R^	(Tropea et al., 2009)
pUC19	pET101 derivative expresses HupX-His_6_, Amp^R^, P_T7_	Invitrogen
pZZ2	pET101 derivative expresses HupZ-His_6,_ Amp^R^, P_T7_	(Sachla et al., 2016)

**Table 2:** Primers.

Prime	Target	Comment	Sequence
ZE685-S	pNZ8008		5’CCCTTGAATTCCACTAGCGTTGCTTTACTG
ZE686-A	pNZ8008		5’GCGCGAAGCTTGGTCCTAAATACTGTTACAG
ZE728-S	*hupZ*		5’CACTCAAAATGATAACACAAGAAATGAAAGAT
ZE729-A	*hupZ*		5’GAGAAGCTTTTAAAATAAGGGTCCTAAATACT
ZE838-S	pUC19		5’GCTGAGATACGCGTAATCATGGTCA
ZE839-A	pUC19		5’ATGGGACAAGCTCGAATTCACTGGC
ZE840-S	pDC123	CM^R^	5’TAGCAATGGTTGCTAACATAGCATTACGG
ZE841-A	pDC123	CM^R^	5’CCAGATTGTACCTAGCGCTCTCATAT
ZE842-S	5’ region of *hupZ*		5’GAGCGCTAGGTACAATCTGGTGCTAAT
ZE843-A	5’ region of h*upZ*		5’ATGATTACGCGTATCTCAGCTATCTTAG
ZE844-S	3’ region of *hupZ*		5’TGAATTCGAGCTTGTCCCATATTGC
ZE845-A	3’ region of *hupZ*		5’CTATGTTAGCAACCATTGCTAATTGG
ZE876-S	pKV111	Adds *Eco*RI to *hupZ::cm^R^*	5’CATAGAATTCATGTGCTGAAGGCGAT
ZE876-A	pKV111	Adds *Eco*RI to *hupZ::cm^R^*	5’CATAGAATTCGTTGTGTGGAATTGTGAGC
ZE978-S	*hupZ*	Adds LIC sequence	5’TACTTCCAATCCAATGCAATGATAACACAAGAAATG
ZE979-A	*hupZ*	Adds LIC sequence	5’TTATCCACTTCCAATGTTATTATTAGTTACTTTCACTGTT

A *hupZ::cm^R^* (chloramphenicol acetyltransferase) allele with flanking regions of chromosome homology was assembled in pUC19 using NEBuilder HiFi Assembly Kit (#E5520) generating plasmid pKV111. The chromosomal *hupZ* upstream region was cloned from GAS strain NZ131 using primers ZE844 and ZE845. The downstream arm using ZE842 and ZE843 and the *cm^R^* gene was amplified from pDC123 using primers ZE840 and ZE841. The *hupZ::cm^R^* allele was amplified from pKV111 with ZE876 and ZE787 primers, adding flanking *Eco*RI sites to move the fragment into the temperature-sensitive vector pJRS700, generating pKV117.

The shuttle vector, pKV138, expressing *hupZ* under GAS *recA* promoter, was generated for complementation. The *hupZ* gene was amplified from NZ131 gDNA and ligated into pLC007. The empty vector control (pKV141) was generated by cutting pLC007 with *Hind*III and self-ligating.

The vector pKV105, expressing *hupZ* under nisin regulation, was used for heterologous expression in *Lactococcus lactis*. The gene was cloned from NZ131 strain using primers ZE685 and ZE686. The insert and pNZ8008 were digested, ligated, and electroporated into MC4100 *E. coli*. Competent MG1363 *L. lactis* that already contained pXL14, which codes the nisin response regulator, were electroporated with either pKV105 or pNZ8008 (as a negative control).

Plasmid pKV102 expressing HupZ with a C-terminal Strep-tag was purchased from Vector Builder. The HupZ sequence was placed in a pET bacterial protein expression vector that contains a T7 promoter and pBR322 origin of replication.

Maltose binding protein (MBP) fusion (pKV130 or pKV135) was generated with ligation-independent cloning into pET/His_6_/MBP/TEV (Addgene plasmid #29656 or pET/MBP/TEV (Addgene plasmid #48311) expression vector as previously described (Porter and Christianson, 2019). Briefly, *hupZ* was cloned from NZ131 with primers ZE978 and ZE979 that added the following upstream, 5’TACTTCCAATCCAATGCA3’, and downstream, 5’TTATCCACTTCAATGTTATTA3’, sequences to the insert. The purified PCR products were incubated with T4 DNA polymerase (Invitrogen 1800-5017), bovine serum albumin (BSA), dithiothreitol (DTT), dCTP, and T4 polymerase buffer. The vector pET/His_6_/MBP/TEV or pET/MBP/TEV was linearized with *Ssp*I restriction enzyme and then incubated with T4 DNA polymerase, BSA, DTT, dGTP, and T4 polymerase buffer. The reactions were cleaned using ethanol precipitation and resuspended in 12 µl of diH_2_O. One microliter of the vector was incubated with 4 µl of insert for 30 min at room temperature. One microliter of 25 mM EDTA was added to the solution and allowed to sit for an additional 15 min and then transformed into *E. coli*.

The GAS Δ*hupZ::cm* ^R^ mutant was generated using insertion inactivation to knock out *hupZ* in the GAS M49 strain NZ131. Competent NZ131 was transformed with the temperature-sensitive pKV117 (harboring the *hupZ::cm^R^* allele and flanking chromosomal region) and plated on THYA with kanamycin (the vector marker) at 30°C. Colonies were then passed three times in THYB with kanamycin at 37°C and were plated with either chloramphenicol or no antibiotic at 30°C. The resulting GAS clones harboring an integrated pKV117 were confirmed by PCR. Daily, individual colonies were propagated at 37°C and screened through replica plating to ensure the loss of the vector marker and the maintenance (knockout mutant) or loss of the *cm^R^* gene (wild-type rescue). The replacing of *hupZ* with *hupZ::cm^R^* allele in M49Lyles or the regeneration of the wild-type *hupZ* allele in M49Rescue was confirmed with PCR.

### Protein Expression

The pKV135 (expressing MBP-HupZ) plasmid was transformed into Invitrogen Chemically Competent BL21(DE3) cells before each expression. All others were grown overnight from a glycerol stock and then diluted in fresh LB media. Cultures were grown at 37°C with 225 rpm until they reached an OD_600_ of 1, induced with 1 mM isopropyl β-D-1-tiogalactopyranosie (IPTG), and incubated overnight at 20°C with 180 rpm. The following day, cells were harvested by centrifugation. HupZ-His_6_- or MBP-HupZ-expressing cells were resuspended in 20 mM Tris (pH 8.0), 100 mM NaCl, and 0.1% Triton X-100. HupZ-Strep cells were resuspended in 20 mM Tris/HCl and 500 mM NaCl. One cOmpleteEDTA-free Protease inhibitor tablet (Roche #1183670001) per 500 ml of grown culture was added before sonification. The cellular debris was pelleted by centrifugation at 20,000 × *g* for 30 min at 4°C, and the lysate was filtered using a 0.22 µM filter unit. Protein was purified on an AKTA FPLC using Cytiva HisTrap HP, MBPTrap HP, or StrepTrap HP Sepharose columns.

**Figure 1:**
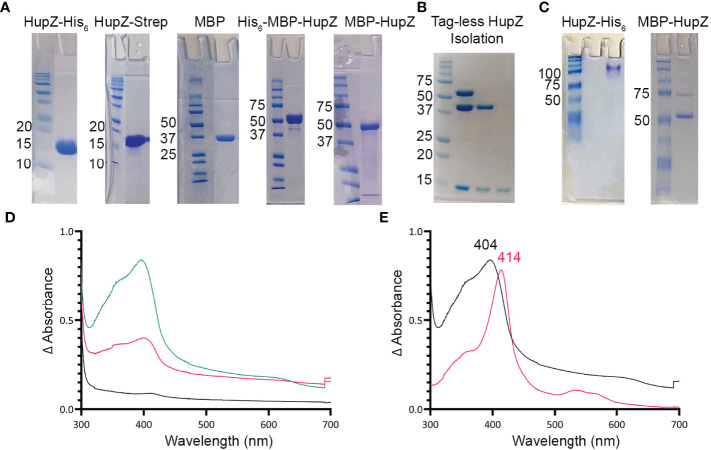
HupZ-Strep binds heme b but without iron coordination. **(A)** SDS-PAGE showing 10 mM of recombinant proteins next to molecular markers. HupZ-His_6_ (18.5 kD) and HupZ-Strep (18.7 kD) were run on 13% acrylamide gel. MBP (40.2 kD) and MBP-HupZ (56.5 kD) were run on 10% acrylamide gels. **(B)** SDS-PAGE (12.5%) showing the cleavage and purification of tag-less HupZ (15 kD). Lane 2 shows partial cleavage, lane 3 shows complete cleavage, and lane 4 is isolated HupZ. **(C)** Native PAGE showing 10 mM of purified proteins next to molecular markers. HupZ-His_6_ (left) or MBP-HupZ (right). **(D)** UV–VIS absorption spectrum of 10 μM HupZ-Strep incubated for 1 h with 5 (pink) or 10 (teal) μM heme. The blank contains reaction buffer with 1% glycerol, 30 mM Arg, and a corresponding amount of heme. **(E)** UV–VIS absorption spectrum of heme bound to HupZ-Strep (black) or HupZ-His6 (pink). Protein samples (10 mM) were incubated with heme for 24 h, free heme was removed by a PD-10 column, and the UV-VIS spectrum was recorded.

Protein purification and size were confirmed using sodium dodecyl sulfate-polyacrylamide gel electrophoresis (SDS-PAGE, **Figure 1A**). Protein concentration was determined by the ThermoScientific Lowry Protein Assay Kit (23240). The buffer used for reconstitutions and degradations consisted of 20 mM sodium phosphate and 500 mM NaCl (pH 7.4). Arginine (30 mM) and 0.1% glycerol were added to the buffer for HupZ-Strep protein to promote solubility.

### TEV Protease Cleavage of His-MBP-HupZ

Purified TEV protease (expressed from pRK793) was generously provided by Dr. Nicholas Noinaj of Purdue University (Tropea et al., 2009). Purified His-MBP-HupZ was incubated with TEV protease at 100 μg/1 μg, respectively, in 200-μl aliquots overnight statically. Cut protein circulated for 1 h at 4°C with resin from three NEBExpress Ni Spin Column rotating head to head in a clean gravity column to remove free His-MBP. Flow-through was collected and subsequently processed *via* an AKTA-FPLC system using Cytiva MBPTrap HP columns to further remove His-MBP. Collected flow-through of tag-free HupZ was concentrated using Amicon^®^ Ultra-15 Centrifugal Filter Unitfilters. Purity was assessed by SDS-PAGE (**Figure 1B**) and quantified by Lowry.

### Mice Mucosal Colonization

Female outbred CD1 (Charles River) mice aged 6 to 8 weeks were used for all experiments. Experiments were performed as previously described (Patras and Doran, 2016; Cook et al., 2018). A day prior to inoculation (day −1), mice were given an intraperitoneal injection of 0.5 mg of β-estradiol valerate (Alfa Aesar) suspended in 100 μl of filter-sterilized sesame oil (Acros Organics MS) to synchronize estrus. On day 0, WT and *ΔhupZ::cm^R^* mutant strains were grown to an OD_600_ = 0.4 and mixed 1:1. Mice were vaginally inoculated with the mixed culture in 10 μl of PBS containing 10^7^ CFU. On days 1, 2, 3, and 5, the vaginal lumen was washed with 50 μl of sterile PBS, using a pipette to gently circulate the fluid approximately 6–8 times. The lavage fluid was then collected and placed on ice for no more than 30 min. Vaginal lavage was serially diluted in PBS and plated on CHROMagar StrepB (WT) or CHROMagar StrepB with chloramphenicol (Δ*hupZ*) plates to obtain CFU counts (Cook et al., 2018). Murine colonization studies were reviewed and approved by Binghamton University Laboratory Animal Resources (LAR) and by the Binghamton Institutional Animal Care and Use Committee (IACUC) under protocols 803-18 and 857-21.

## Results

### Recombinant HupZ Proteins Expressed Without a His_6_ Tag Binds Heme b

We previously showed that a recombinant HupZ protein containing a C-terminal fusion to His_6_ tag (HupZ-His_6_) binds and degrades heme *in vitro* (Sachla et al., 2016). HupZ-His_6_ crystalized as a homodimer with a split β-barrel conformation, a fold also seen in FMN-binding heme-degrading proteins described in several bacterial species (Sachla et al., 2016; Lyles and Eichenbaum, 2018). Additional investigations revealed that this recombinant HupZ protein interacts with heme *in vitro* by its His_6_ tag, leading to a higher-order oligomeric structure, heme stacking, and degradation (Traore et al., 2021). These findings cast doubts about the function and the role of HupZ in heme metabolism. To reexamine heme-binding by HupZ, we constructed a new recombinant C-terminal fusion replacing the His_6_ with a Step-tag (HupZ-Strep) to facilitate purification. We expressed and purified the recombinant HupZ-Strep (**Figure 1A**), but the purified protein was not stable and precipitated out of the solution. We added 1% glycerol and 30 mM arginine to the buffer to increase stability for later heme titration experiments (Vagenende et al., 2009; Kudou et al., 2011). Titration of HupZ-Strep with externally added heme revealed the formation of a growing UV-VIS absorption peak at 404 nm that is indicative of heme-binding (**Figure 1B**). However, the heme bound form of HupZ-Strep exhibited a shift in absorption maxima compared to the holo HupZ-His_6_, which has a 414-nm Soret peak (**Figure 1C**). Additionally, unlike HupZ-His_6_, the absorption spectrum of HupZ-Strep did not include the α and β bands between 500 and 600 nm, implying HupZ-Strep binds heme without an axial heme ligand that coordinates the iron (Giovannetti, 2012).

**Figure 2:**
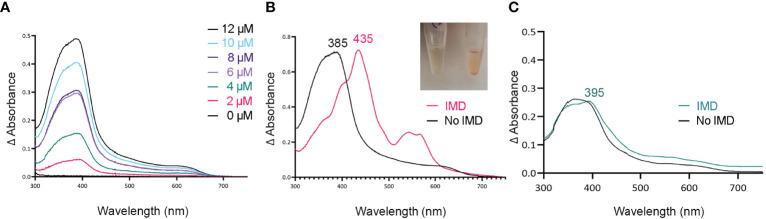
Tag-less HupZ requires the imidazole group to coordinate heme b iron. UV-VIS absorption spectra of 10 μM tag-less HupZ **(A)** incubated for 1 h with a range of heme concentrations. The blank contained the reaction buffer and equivalent concentration of heme. Tag-less HupZ was incubated with 20 μM heme for 24 h, free heme was removed with PD-10 column (**B**, black). Then 1.6 mM imidazole (IMD, pink) was added to the cuvette. This addition causes the solution in the test tubes to change to pink (Insert). Lastly **(C)**, the spectra for 10 μM of heme was taken in the standard reaction buffer (black) and with the addition of 1.6 mM IMD (teal). The blank contained only the reaction buffer.

The crystal structure of HupZ-His_6_ indicates that the protein’s C-terminus is close to where the HupZ dimers form a quaternary β-barrel (Sachla et al., 2016). To avoid the possibility that a C-terminal addition may interfere with the function and stability of HupZ, we constructed N-terminal fusions to two different MBP-containing vectors (His-MBP-HupZ and MBP-HupZ) and purified the proteins (**Figure 1A**). We generated a tag-less HupZ by cleaving His-MBP-HupZ with TEV protease (**Figure 1B**), leaving a serine residue after cleavage. We assayed heme binding by incubating 10 μM of tag-less HupZ with increasing heme concentration (**Figure 2A**). To confirm heme binding, the protein was allowed to set with 2× concentration of heme overnight and then passed through a PD-10 desalting column to remove any upbound heme (**Figure 2B**). Overall, tag-less HupZ exhibited a heme-binding spectrum similar to HupZ-Strep and different from HupZ-His_6_ (**Figures 1D, E**). Tag-less HupZ’s Soret was broad and peaked at 385 nm. The 500- through 600-nm range lacked the α and β bands that indicated coordination of the central heme-iron.

**Figure 3:**
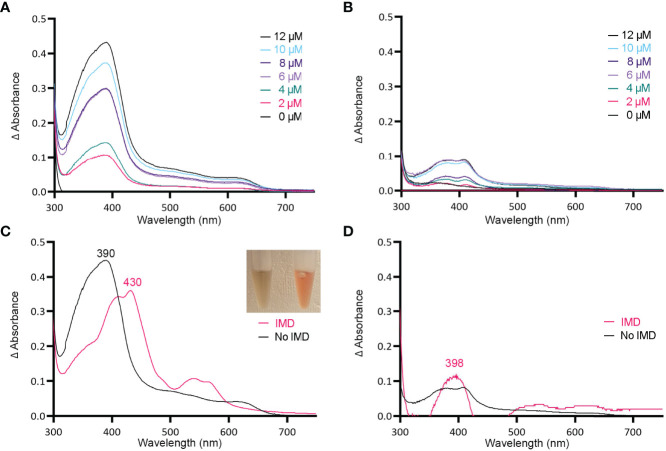
MBP-HupZ binds heme b and uses exogenous histidine for iron coordination. UV-VIS absorption spectra of 10 μM MBP-HupZ **(A)** or MBP **(B)** incubated for 1 h with a range of heme concentrations. The blank contained the reaction buffer and equivalent concentration of heme. MBP-HupZ was incubated with 20 μM heme for 24 h, free heme was removed, and the UV-VIS spectrum was determined before (**C**, black) or after the addition of 1.6 mM imidazole (IMD, pink). MBP (10 μM) was incubated with 20 μM heme for 24 h, free heme was removed, and the UV-VIS spectrum was determined before (**D**, black) or after the addition of IMD (pink).

Tag-less HupZ was not highly stable and prone to precipitate. Due to the solubility problems, further testing used a fusion to MBP (MBP-HupZ), which often aids in protein solubility. Unlike HupZ-Strep and tag-less HupZ, MBP-HupZ is soluble. Heme titration and reconstitution experiments demonstrated that the holo MBP-HupZ’s UV-VIS spectrum (**Figures 3A, C**) is similar to heme binding by tag-less HupZ. We also assess the heme-binding of a purified MBP protein as a negative control. MBP bound only a negligible amount of heme, exhibiting a vastly different absorption spectrum during heme titrations and reconstitutions (**Figures 3B, D**). MBP-HupZ also migrated as a monomer on native PAGE, indicating that it does not assemble into a high oligomeric state *in vitro* like HupZ-His_6_ (**Figure 1C**) (Traore et al., 2021). Therefore, all three recombinant HupZ proteins bind heme, albeit without an axial heme ligand. These findings suggest that heme-binding is native to the HupZ protein and independent of the tag, the location of the fusion, or the multimeric state.

### Tag-Less and MBP-HupZ Use an Exogenous Histidine for Iron Coordination Of Heme b

Histidine is a common residue in short peptides that bind heme and often functions as the heme axial ligand in hemoproteins (Wissbrock et al., 2019). The imidazole moiety of histidine interacts with iron and other transition metals during binding. Since holo tag-less and MBP-HupZ did not exhibit iron coordination, we tested if exogenous imidazole could serve this function *in vitro* (**Figures 2B** and **3C**). In both recombinant proteins, the addition of 1.6 mM imidazole caused a shift in the Soret peak, the generation of α and β bands, and the protein solution turned red (**Figures 2B** inset and **3C** inset). As a control, we also measured the UV-VIS spectrum of MBP after incubation with heme, which exhibited minor spectral changes and still lacked α and β bands (**Figure 3D**). The UV-VIS spectra of free heme did not change with the addition of imidazole (**Figure 2C**). These observations suggest that exogenous imidazole can coordinate the iron in holo tag-less and MBP-HupZ but not in MBP or free heme. Together, this indicates that HupZ binds heme without an axial heme ligand but can readily interact with an exogenous imidazole group to coordinate the iron.

### MBP-HupZ Degrades Heme b in the Presence of an Exogenous Imidazole

**Figure 4:**
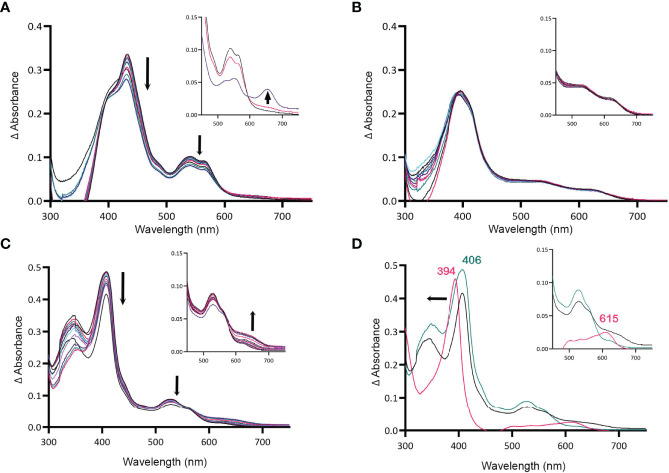
MBP-HupZ degrades heme with the exogenous imidazole group. UV-VIS absorption spectra of 10 μM heme b bound to MBP-HupZ with 1.6 mM imidazole **(A)** or without **(B)** incubated with 10 μM ferredoxin, NADPH, an NADPH regeneration system, and catalase. After acidification of the MBP-HupZ reaction with imidazole, a peak at 660 nm formed (**A,** inset, purple). The blank contained 10 μM ferredoxin, NADPH, an NADPH regeneration system, and catalase, with or without 1.6 mM imidazole. MBP-HupZ (10 μM) in solution with 10 μM MP11, 10 μM ferredoxin, NADPH, an NADPH regeneration system, and catalase **(C)**. **(D)** The zero minute of the MP11 (teal) and the 6-h time point (black). After acidification of the reaction (pink), the Soret shifted from 406 to 394 nm, and a peak formed at 626 nm (**D**, inset, pink). The blank contained 10 μM ferredoxin, an NADPH regeneration system, and catalase, with or without 1.6 mM imidazole.

HupZ-His_6_ degrades heme *in vitro*, releasing CO, free iron, and a chromophore (Sachla et al., 2016). Since externally added imidazole can coordinate the heme iron in holo-MBP-HupZ, we tested if MBP-HupZ can also break down the heme under these conditions. Holo-MBP-HupZ was incubated with 1.6 mM imidazole, ferredoxin (as a reducing agent), NADPH, an NADPH regeneration system (glucose-6-phosphate and glucose-6-phosphate dehydrogenase), and catalase (to control for non-enzymatic degradation of heme by hydrogen peroxide) and allowed to run for 6 h (**Figure 4A**). As indicated by the arrows, the Soret and the α and β bands decreased steadily during incubation, indicating heme degradation (**Figure 4B**). Still, the MBP-HupZ reaction did not result in an absorption peak at 600–700 mm, indicating the formation of biliverdin or similar molecules. *In vitro* heme degradation by some heme oxygenases (e.g., HemO and HemO/PigA) can result in ferric-biliverdin, which has no absorption properties (Zhu et al., 2000; Ratliff et al., 2001). To liberate the iron, we treated the MBP-HupZ reaction with acid. As with HemO, the reaction acidification caused the reduction of the α and β bands and formed a chromophore, although at 660 nm and not 680 nm as with HemO. Hence, holo MBP-HupZ can degrade heme only when an externally provided imidazole group is present to coordinate the iron. No significant spectral changes were observed in the absence of imidazole (**Figure 4B**), indicating that holo-MBP-HupZ did not degrade the heme.

### HupZ Showed Weak Degradation of Heme c (MP11)

All of the heme-degrading proteins described to date catalyze the breakdown of heme b. The one exception is Pden_1323, from *Paracoccus denitrificans*, who also belongs to the HugZ family. Pden_1323 lacks the C-terminal loop that contains the axial heme ligand (His245 in HugZ) but degrades heme c bound to a cytochrome fragment (MP11) (Li et al., 2021). In cytochrome c, the heme iron is coordinated by a histidine in proteaceous region attached to heme c and that histidine is retained in MP11 (Kranz et al., 2009). Since MBP-HupZ can degrade heme if the iron is coordinated by exogenous imidazole, we also tested if it could degrade heme c provided by MP11. MBP-HupZ bound MP11 with a 406-nm Soret and had α and β bands (**Figure 4D**). MBP-HupZ bound to MP11 was then tested for degradation with ferredoxin, NADPH, and NADPH regeneration system (in the presence of catalase). The reaction resulted in a progressive, though limited, decrease in the Soret and the α and β bands (**Figure 4C**). Like with heme b, the HupZ reaction did not produce an absorption peak between 600 and 700 mm. However, subsequent acidification of the solution led to forming a 615-nm chromophore (**Figure 4D**). Together, the data show that using ferredoxin, HupZ only moderately degrades heme b and heme c *in vitro*. It is possible that the fusion with MBP may hinder the degradation or *in vitro* conditions are not optimal.

### Heme c Does Not Appear to Serve as an Iron Source for GAS

**Figure 5:**
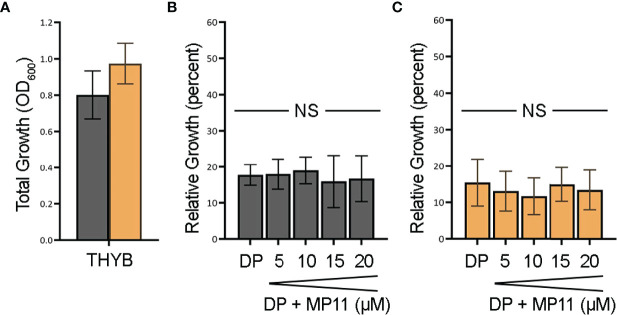
GAS cannot use MP11 as an iron source. Overnight growth of GAS wild-type rescue (gray) and *ΔhupZ* (orange) strains in THYB **(A)**. Relative growth of GAS wild-type rescue THYB-DP with a range of MP-11 compared to THYB growth **(B)**. Relative growth Δ*hupZ* under the same conditions **(C)**. No significance was determined using the Student’s *t*-test. NS, not significant.

To test if the observed heme c *in vitro* degradation by HupZ is biologically relevant, we constructed a Δ*hupZ* mutant in GAS by insertion inactivation and assessed the ability of both the wild-type rescue and Δ*hupZ* strains to use a fragment of heme c (MP11) as an iron source (**Figure 5**). Inactivation of *hupZ* had a small positive impact on growth in the regular laboratory THYB (**Figure 5A**). Adding the iron chelator dipyridyl to THYB (THYB-DP) restricted growth in both strains to less than 20% and supplementing the medium with a range of 5–20 μM of MP11 could not significantly restore growth in either strain of bacteria, as indicated by a 1-way ANOVA across treatment types within each strain. There was also no significant difference between wild-type and Δ*hupZ* strains when comparing identical treatment conditions using a Student’s *t*-test (**Figure 5**). A 2-way ANOVA covering aggregated data points for wild-type percentage growth to aggregated Δ*hupZ* percentage growth indicated that there was a significant mean difference in the wild type compared to the mutant in the iron-depleted media (17.5% to 14.2%, respectively). Indicating that while GAS does not utilize MP11 as an iron source, HupZ may provide relief from heme c toxicity.

### HupZ Contributes to Heme b Metabolism *In Vivo*

**Figure 6:**
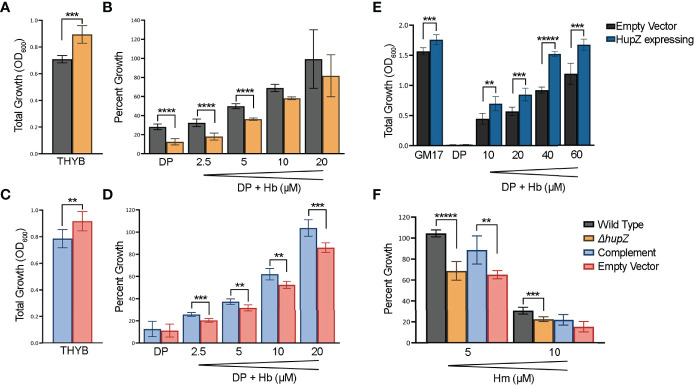
HupZ contributes to bacterial growth on heme b-iron and aids GAS in heme b tolerance. Overnight growth of GAS wild-type rescue (gray) and Δ*hupZ* (orange) strains in THYB **(A)**. Relative growth of GAS wild-type rescue and Δ*hupZ* strains in THYB-DP with a range of hemoglobin (Hb) compared to THYB growth **(B)**. Overnight growth of complement (light blue) and empty vector (pink) in THYB **(C)**. Relative growth of GAS Δ*hupZ* strain with HupZ expressing (light blue, complement) or empty vector (pink) in THYB-DP with a range of HB **(D)**. Overnight growth of *L. lactis* in GM17 **(E)**, GM17-DP, or GM17-DP and a range of Hb, with either an empty (black) or HupZ expressing vector (dark blue). Relative growth of GAS wild-type rescue, complement, and empty vector strains in THYB with either 5 or 10 μM heme (Hm) compared to normal THYB growth **(F)**. The data represent three independent experiments and were analyzed using the Student’s *t*-test, where ** indicates a P-value > 0.001, *** > 0.0001, **** > 0.00001, and ***** > 0.000001.

To further evaluate the role of HupZ in heme use *in vivo*, we examined the mutant and the wild-type rescue strain for heme use and sensitivity. Unlike with MP11, supplementation of THYB-DP with hemoglobin at a range of 2.5 to 20 μM restored GAS growth in THYB-DP, indicating that both strains can use hemoglobin as an iron source (**Figures 6A, B**). The hemoglobin dose–response was delayed in the Δ*hupZ* strain compared to the wild-type rescue strain. For complementation, we expressed *hupZ* in trans from GAS *recA* promoter. Comparing the complemented and control (empty vector) strains revealed a small but statistically significant difference between the strains when grown on hemoglobin iron (**Figures 6C, D**). First, data show that losing *hupZ* reduces GAS’ ability to use heme iron.

The moderate phenotype of the *hupZ* mutant suggests redundancy in mechanisms to gain iron by GAS. Hence, we also tested the influence of *hupZ* expression on hemoglobin iron use by a heterologous host. HupZ was expressed from the P_nis_ promoter in *L. lactis* (**Figure 6E**). Lactococcal growth in GM17 was inhibited with 10 mM DP. Adding hemoglobin to the iron-restricted medium restored growth, indicating that *L. lactis* can use hemoglobin as an iron source. *L. lactis* expressing *hupZ* responds to a lower hemoglobin concentration and reaches a higher maximal density than the *L. lactis* harboring an empty vector. Second, HupZ promotes the use of heme iron in both GAS and *L. lactis*.

We tested the sensitivity of the GAS mutant and the wild-type rescue strains to free heme (**Figure 6F**). The addition of 5 mM heme to THYB restricted the growth of the *hupZ* mutant while it had no impact on the rescue strain. The addition of 10 μM heme inhibited the growth of both strains, but the wild-type rescue strain grew better than the *hupZ* mutant. Third, *hupZ* helps GAS manage heme toxicity at a low heme concentration.

### Loss of *hupZ* Decreases the Fitness of GAS in a Mucosal Colonization Competition

**Figure 7:**
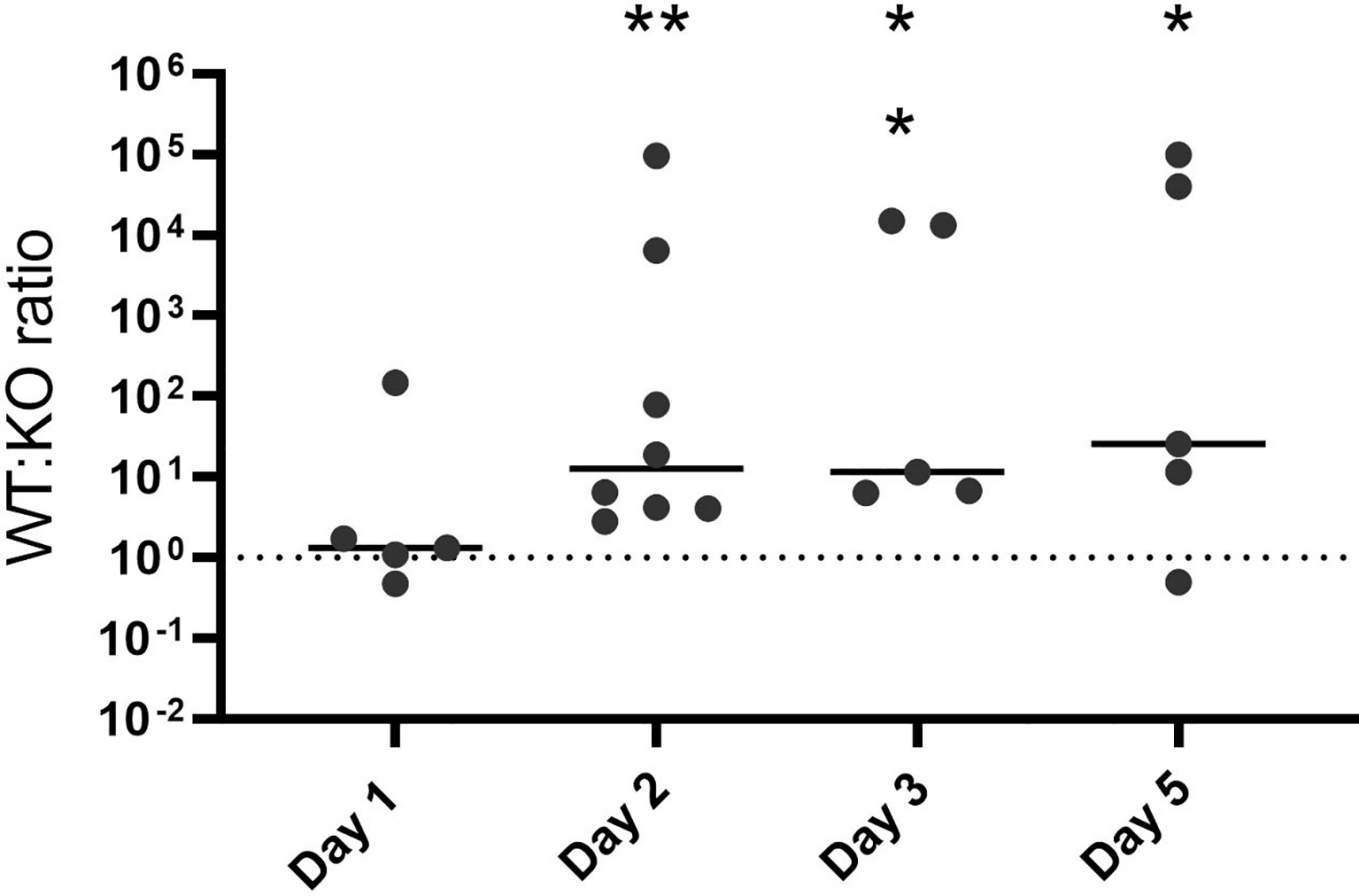
HupZ aids GAS fitness in mucosal colonization model. Mixtures (1:1) of WT and Δ*hupZ* mutant cells were inoculated intravaginally into mice on day 0. Vaginal lavage samples taken at days 1, 2, 3, and 5 post-inoculation were plated to determine the competitive index of WT:Δ*hupZ* mutant cells recovered. By day 2 and through to day 5, significantly fewer Δ*hupZ* mutant cells were recovered compared to WT. The data represent two independent experiments and were analyzed using the Kruskal–Wallis test of ratios, where * indicates a *p*-value > 0.01 and ** > 0.001.

Colonization of the host mucosa constitutes an important first step in GAS pathogenesis *in vivo* (Walker et al., 2014; Zhu et al., 2020). We used a murine colonization model to determine whether *hupZ* plays a role in the ability of GAS to colonize the vaginal mucosa. When wild-type rescue and Δ*hupZ* mutant cells were mixed at a 1:1 ratio and inoculated intravaginally into mice, they were initially able to colonize in approximately equal ratios (**Figure 7**). Following the initial vaginal lavage on day 1, the mutants were no longer able to compete with the WT cells, and the ratio of recovered WT:Δ*hupZ* mutant cells increased dramatically. The data indicate that *hupZ* is important in allowing GAS to maintain host surface colonization.

## Discussion

Heme acquisition and iron release are vital for GAS infections, as they supply the pathogen with growth-essential iron in the host environment. GAS lacks genes that share sequence homology to canonical heme oxygenases, and hence how this pathogen processes heme to release the iron is an enigma. We hypothesized that HupZ, a small and presumably cytoplasmic protein, is involved in heme metabolism because it is regulated by MtsR and is co-expressed with the heme receptor, *hupY*. The crystal structure of HupZ-His_6_ is similar to the heme-degrading enzyme HugZ, particularly on the split barrel fold of the C-terminal domain (Sachla et al., 2016). HupZ, however, lacks the N-terminal domain. Moreover, *in vitro* analysis revealed that a recombinant HupZ-His_6_ binds and degrades heme *in vitro*. Holo-HupZ-His_6_ exhibits a UV-VIS spectrum with a prominent Soret and α and β bands in the 500–600 nm range indicative of heme bound with an axial heme ligand (Sachla et al., 2016). Resonance Raman spectroscopy indicated that the axial heme ligand in HupZ-His_6_ was a histidine residue, but replacing the only native HupZ histidine residue with alanine did not affect the spectrum (Traore et al., 2021). These observations suggest that one of the six histidine residues in the purification tag interacts in the heme-binding capacity of the recombinant HupZ-His_6_ protein.

In this study, we examined HupZ’s function using biochemical and genetic approaches. Using new recombinant HupZ proteins with a carboxy-terminal fusion to a Strep-tag (HupZ-Strep), a tag-less HupZ, or an amino-terminal fusion to MBP (MBP-HupZ), we demonstrated that HupZ binds heme independently of the tag nature or location (**Figures 1**–**3**). Interestingly, without the His_6_ tag, MBP-HupZ does not appear to assemble into the high oligomeric state exhibited by the HupZ-His_6_ variant, (**Figure 1C**). This state is thought to promote heme degradation by HupZ-His_6_ (Traore et al., 2021). More work, however, is needed to determine the oligomeric state of native HupZ. While the spectrum indicates that HupZ lacks a native axial heme ligand, tag-less and MBP-HupZ exhibited iron coordination provided by free imidazole (**Figures 2** and **3**) or heme c (**Figure 4**).

As we see with HupZ, the protein framework can provide sufficient interactions for binding within a relatively heme-specific binding pocket. Historically, it was presumed that the bond(s) between the heme iron and the amino acid(s) coordinating the iron is the major force holding the heme into the protein. However, experiments on globin and cytochrome mutants in which the proximal histidine was changed to glycine and the side chain was replaced by an imidazole showed that the protein could still incorporate heme even without a coordinate/covalent bond attachment (Schneider et al., 2007). Additionally, mutation of the HugZ axial heme ligand, His245, to alanine, glutamine, or asparagine, could all degrade heme, indicating that the side chain of the residues was not required for enzymatic degradation and may serve a role in the recognition and binding specificity of heme (Hu et al., 2011). Indeed, when the axial heme ligand (His209) of the heme shuttle protein, PhuS, of *P. aeruginosa* is mutated, the *in vitro* protein can coordinate the iron with neighboring His210 or His212 instead (Tripathi et al., 2013).

MBP-HupZ with heme-iron coordinated in the presence of a reducing partner exhibited a weak decrease in the Soret and the α and β band (**Figure 4**). Heme degradation enzymes require reduction partners that provide the electrons necessary for degradation. Cytochrome P450 reductase is the native reducing partner of the mammalian HO-1. The native partners of most bacterial heme-degrading enzymes are not known. The only exceptions are the ferredoxin reductase FPR in *P. aeruginosa* and the reductases IruO and NtrA in *S. aureus*, which facilitate the reaction of HemO/PigA and IsdG/I, respectively (Wang et al., 2007; Hannauer et al., 2015). Heme degradation with native reducing partners results in forming a linear porphyrin (e.g., biliverdin) and free iron. The HupZ degradation reaction, using ferredoxin as a reducing partner, results in a product that does not have spectral properties. The formation of a chromophore that absorbed at 660 nm after acidification (**Figure 4D**, inset) suggests that the HupZ reaction stopped after the formation of ferric-biliverdin (or a similar molecule), which is missing a spectroscopic signature. Similar observations were made with the heme-degrading enzyme PigA/HemO of *P. aeruginosa*, and HemO of *N. meningitidis*, as well as the oxidoreductive cleavage of verdohemochrome IX-α (Saito and Itano, 1982; Zhu et al., 2000; Ratliff et al., 2001).

MBP-HupZ-bound MP11 spectra contained α and β bands consistent with coordination of the heme iron and limited degradation to a 615-nm chromophore over 6 h (released by acidification, **Figure 4D**). Hence, HupZ weakly degrades heme c *in vitro*. HupZ’s dependency on exogenous imidazole and heme c degradation are reminiscent of Pden_1323, the only heme c-degrading enzyme to be described. Still, Pden_1323 fully oxygenates MP11 in 5 min while HupZ facilitates only partial (~0.2–0.4) turnover in 6 h. The weak degradation activity could result from the absence of the native reducing partner or the MBP fusion, or it is not physiologically relevant. To assess the physiological relevance, we tested the ability of WT NZ131 and a Δ*hupZ* strain to use the heme c fragment, MP11, as an iron source but did not see notable growth (**Figure 5**). Pden_1323 and HupZ create a new subgroup in the FMN-binding class of heme-binding or -degrading enzymes. While sharing overall homology with the HugZ protein family, they both lack the C-terminal loop that typically contains the axial heme ligand and the N-terminal α/β domain. This omission creates a larger opening where the heme-binding pocket is believed to be based, allowing Pden_1323 and HupZ to accommodate the larger heme c ligand. Interestingly, HugZ N-terminus is not required for heme degradation; in fact, a C-terminal domain truncated mutant of HugZ demonstrated an increased rate of degradation compared to full-length HugZ (Hu et al., 2011).

*In vivo*, the loss of *hupZ* results in a decrease in heme b iron use by GAS at low concentrations (**Figure 6B**). The increase in growth on hemoglobin iron exhibited by *L. lactis* expressing *hupZ* provides additional support and *hupZ* contribution to heme b metabolism (**Figure 6E**). Together, these observations are consistent with a function as a heme chaperone.

Transcriptome analysis of GAS during murine vaginal carriage showed that the *hupYZ* operon is significantly upregulated, exhibiting a 27- and 37-fold increase in expression of *hupY* and *hupZ*, respectively, compared with bacteria grown in a chemically defined medium (Cook et al., 2019). We tested the *hupZ* mutant in a murine mucosal colonization competition (**Figure 7**). While the ratio of wild-type rescue to mutant was equal on the first day, by the second day, there were significantly fewer *hupZ* mutant cells recovered than wild-type rescue, a trend that continued through to the 5 days. In all, this indicates that *hupZ* promotes bacterial fitness in the host.

Lastly, polyhistidine tags are one of the most widely used for protein purification. Often the tags are removed after purification by inserting a protease recognition sequence. However, low reaction efficiency coupled with the requirement of costly enzymes and an additional purification step means that they are commonly retained (Jenny et al., 2003; Kielkopf et al., 2021). Yet, as indicated through this work, the utilization of a His_6_ tag may be detrimental to determining the function of a protein, as the histidine residue may interact with an unintended epitope. The retention of these tags during experimental conditions can also change ligand binding dynamics. The two-dimensional infrared vibrational echo spectroscopy of His_6_-myoglobin showed a significant change in the short time scale dynamics of binding carbon monoxide compared to native myoglobin, although there was no effect on the UV/VIS spectra (Thielges et al., 2011). The terminal placement (i.e., amino or carboxy) affects the product regiospecificity of the carbonyl reductase S1 from *Candida magnoliae* (Haas et al., 2017). With HupZ-His_6_, the tag not only interacted with the bound heme but also promoted higher oligomeric states. Given histidine’s propensity to affect protein dynamics and regiospecificity, along with their prominences in heme-binding pockets, they may not be suitable for heme-binding studies.

In summary, HupZ binds heme b and heme c and relies on exogenous imidazole for degradation *in vitro*. GAS mutants lacking *hupZ* can use heme iron albeit less efficiently. These observations, combined with the low turnover in heme b and MP-11 *in vitro* degradations, suggest that *in vivo* HupZ is likely a heme chaperone, or it contributes to heme detoxification by a yet-to-be-defined mechanism.

## Data Availability Statement

The raw data supporting the conclusions of this article will be made available by the authors, without undue reservation.

## Ethics Statement

The animal study was reviewed and approved by Binghamton University Laboratory Animal Resources (LAR) and by the Binghamton Institutional Animal Care and Use Committee (IACUC) under protocols 803-18 and 857-21.

## Author Contributions

LT conducted the mouse model under the direction of LC. CO generated the tag-less HupZ and KL conducted the remaining experiments, both under the supervision of ZE. KL generated the figures and both she and ZE contributed equally to the generation of the manuscript. All authors contributed to the article and approved the submitted version.

## Funding

KL received a Fellowship through the Georgia State University Molecular Basis of Disease program.

## Conflict of Interest

The authors declare that the research was conducted in the absence of any commercial or financial relationships that could be construed as a potential conflict of interest.

## Publisher’s Note

All claims expressed in this article are solely those of the authors and do not necessarily represent those of their affiliated organizations, or those of the publisher, the editors and the reviewers. Any product that may be evaluated in this article, or claim that may be made by its manufacturer, is not guaranteed or endorsed by the publisher.

## References

[B1] Barnett T. C. Bowen A. C. Carapetis J. R. (2018). The Fall and Rise of Group A Streptococcus Diseases. Epidemiol. Infect. 147, E4. doi: 10.1017/S0950268818002285 30109840 PMC6518539

[B2] Bates C. S. Montanez G. E. Woods C. R. Vincent R. M. Eichenbaum Z. (2003). Identification and Characterization of a Streptococcus Pyogenes Operon Involved in Binding of Hemoproteins and Acquisition of Iron. Infect. Immun. 71 (3), 1042–1055. doi: 10.1128/IAI.71.3.1042-1055.2003 12595414 PMC148835

[B3] Bates C. S. Toukoki C. Neely M. N. Eichenbaum Z. (2005). Characterization of MtsR, a New Metal Regulator in Group A Streptococcus, Involved in Iron Acquisition and Virulence. Infect. Immun. 73 (9), 5743–5753. doi: 10.1111/j.1365-2958.2010.07157.x 16113291 PMC1231137

[B4] CDC. Centers for Disease Control and Prevention (2019). Active Bacterial Core Surveillance Report, Emergin Infections Program Network, Group A Streptococcus, 2019. Available at: www.cdc.gov/abcs/downloads/GAS_Surveillance_Report_2019.pdf.

[B5] Chaffin D. O. Rubens C. E. (1998). Blue/white Screening of Recombinant Plasmids in Gram-Positive Bacteria by Interruption of Alkaline Phosphatase Gene (phoZ) Expression. Gene (1998) 219 (1-2), 91–9. doi: 10.1016/s0378-1119(98)00396-5 9757005

[B6] Chatterjee N. Cook L. C. C. Lyles K. V. Nguyen H. A. T. Devlin D. J. Thomas L. S.. (2020). Novel Heme Transporter From the Energy Coupling Factor Family Is Vital for Group A Streptococcus Colonization and Infections. A. J. Bacteriol. 202 (14), e000205-20. doi: 10.1128/JB.00205-20 PMC7317044 32393520

[B7] Cook L. C. C. Chatterjee N. Li Y. Andrade J. Federle M. J. Eichenbaum Z. (2019). Transcriptomic Analysis of Streptococcus Pyogenes Colonizing the Vaginal Mucosa Identifies Hupy, an MtsR-Regulated Adhesin Involved in Heme Utilization. mBio. 10 (3), e00848-19. doi: 10.1128/mBio.00848-19 31239377 PMC6593403

[B8] Cook L. Hu H. Maienschein-Cline M. Federle M. (2018). A Vaginal Tract Signal Detected by the Group B Streptococcus SaeRS System Elicits Transcriptomic Changes and Enhances Murine Colonization. Infect. Immun. 86 (4), e00762–e00717. doi: 10.1128/IAI.00762-17 29378799 PMC5865029

[B9] Davies M. R. Holden M. T. Coupland P. Chen J. H. Venturini C. Barnett T. C.. (2015). Emergence of Scarlet Fever Streptococcus Pyogenes Emm12 Clones in Hong Kong is Associated With Toxin Acquisition and Multidrug Resistance. Nat. Genet. 47 (1), 84–87. doi: 10.1038/ng.3147 25401300

[B10] de Ruyter P. G. Kuipers O. P. de Vos W. M. (1996). Controlled Gene Expression Systems for Lactococcus Lactis With the Food-Grade Inducer Nisin. Appl. Environm. Microbiol., 62(10), 3662–3667. doi: doi: 10.1128/aem.62.10.3662-3667.1996 PMC168174 8837421

[B11] Giovannetti R. (2012). “The Use of Spectropotometry UV-Vis for the Study of Porphyrins,” in Macro to Nano Spectroscopy. Ed. Uddin J. (Croatia: InTech).

[B12] Haas J. Hackh M. Justus V. Muller M. Ludeke S. (2017). Addition of a Polyhistidine Tag Alters the Regioselectivity of Carbonyl Reductase S1 From Candida Magnoliae. Org. Biomol. Chem. 15 (48), 10256–10264. doi: 10.1039/C7OB02666H 29182182

[B13] Hannauer M. Arifin A. J. Heinrichs D. E. (2015). Involvement of Reductases IruO and NtrA in Iron Acquisition by Staphylococcus Aureus. Mol. Microbiol. 96 (6), 1192–1210. doi: 10.1111/mmi.13000 25777658

[B14] Hu Y. Jiang F. Guo Y. Shen X. Zhang Y. Zhang R.. (2011). Crystal Structure of HugZ, a Novel Heme Oxygenase From Helicobacter Pylori. J. Biol. Chem. 286 (2), 1537–1544. doi: 10.1074/jbc.m110.172007 21030596 PMC3020762

[B15] Jenny R. J. Mann K. G. Lundblad R. L. (2003). A Critical Review of the Methods for Cleavage of Fusion Proteins With Thrombin and Factor Xa. Protein Expr. Purif. 31 (1), 1–11. doi: 10.1016/s1046-5928(03)00168-2 12963335

[B16] Kielkopf C. L. Bauer W. Urbatsch I. L. (2021). Expressing Cloned Genes for Protein Production, Purification, and Analysis. Cold Spring Harb. Protoc. 2021 (2), 43–69. doi: 10.1101/pdb.top102129 33272973

[B17] Kleerebezem M. Beerthuyzen M. M. Vaughan E. E. De Vos W. M. Kuipers O. P. (1997). Controlled Gene Expression Systems for Lactic Acid Bacteria: Transferable Nisin-Inducible Expression Cassettes for Lactococcus, Leuconostoc, and Lactobacillus Spp. Applied and Environmental Microbiology. (1997) 63 (11), 4581–4. 10.1128/aem.63.11.4581-4584.1997 PMC168776 9361443

[B18] Kranz R. G. Richard-Fogal C. Taylor J. S. Frawley E. R. (2009). Cytochrome C Biogenesis: Mechanisms for Covalent Modifications and Trafficking of Heme and for Heme-Iron Redox Control. Microbiol. Mol. Biol. Rev. 73 (3), 510–528. doi: 10.1128/MMBR.00001-09 19721088 PMC2738134

[B19] Kudou M. Yumioka R. Ejima D. Arakawa T. Tsumoto K. (2011). A Novel Protein Refolding System Using Lauroyl-L-Glutamate as a Solubilizing Detergent and Arginine as a Folding Assisting Agent. Protein Expr. Purif. 75 (1), 46–54. doi: 10.1016/j.pep.2010.08.011 20817098

[B20] Li S. Isiorho E. A. Owens V. L. Donnan P. H. Odili C. L. Mansoorabadi S. O. (2021). A Noncanonical Heme Oxygenase Specific for the Degradation of C-Type Heme. J. Biol. Chem. 296, 100666. doi: 10.1016/j.jbc.2021.100666 33862082 PMC8131568

[B21] Lyles K. V. Eichenbaum Z. (2018). From Host Heme To Iron: The Expanding Spectrum of Heme Degrading Enzymes Used by Pathogenic Bacteria. Front. Cell Infect. Microbiol. 8, 198. doi: 10.3389/fcimb.2018.00198 29971218 PMC6018153

[B22] Marchetti M. De Bei O. Bettati S. Campanini B. Kovachka S. Gianquinto E.. (2020). Iron Metabolism at the Interface Between Host and Pathogen: From Nutritional Immunity to Antibacterial Development. Int. J. Mol. Sci. 21 (6), 1–44. doi: 10.3390/ijms21062145 PMC7139808 32245010

[B23] Patras K. A. Doran K. S. (2016). A Murine Model of Group B Streptococcus Vaginal Colonization. J. Vis. Exp. 117 (117), 54708. doi: 10.3791/54708 PMC5226234 27911391

[B24] Porter N. J. Christianson D. W. (2019). Preparation of a New Construct of Human Histone Deacetylase 8 for the Crystallization of Enzyme-Inhibitor Complexes. Methods Enzymol. 626, 561–585. doi: 10.1016/bs.mie.2019.06.029 31606091 PMC6941479

[B25] Ratliff M. Zhu W. Deshmukh R. Wilks A. Stojiljkovic I. (2001). Homologues of Neisserial Heme Oxygenase in Gram-Negative Bacteria: Degradation of Heme by the Product of the pigA Gene of Pseudomonas Aeruginosa. J. Bacteriol. 183 (21), 6394–6403. doi: 10.1128/JB.183.21.6394-6403.2001 11591684 PMC100135

[B26] Sachla A. J. Ouattara M. Romero E. Agniswamy J. Weber I. T. Gadda G.. (2016). *In Vitro* Heme Biotransformation by the HupZ Enzyme From Group A Streptococcus. Biometals. 29 (4), 593–609. doi: 10.1007/s10534-016-9937-1 27154580

[B27] Saito S. Itano H. A. (1982). Verdohemochrome IX Alpha: Preparation and Oxidoreductive Cleavage to Biliverdin IX Alpha. Proc. Natl. Acad. Sci. U. S. A. 79 (5), 1393–1397. doi: 10.1073/pnas.79.5.1393 6951184 PMC345979

[B28] Schmitt M. P. (1997). Utilization of Host Iron Sources by Corynebacterium Diphtheriae: Identification of a Gene Whose Product is Homologous to Eukaryotic Heme Oxygenases and is Required for Acquisition of Iron From Heme and Hemoglobin. J. Bacteriol. 179 (3), 838–845. doi: 10.1128/jb.179.3.838-845.1997 9006041 PMC178768

[B29] Schneider S. Marles-Wright J. Sharp K. H. Paoli M. (2007). Diversity and Conservation of Interactions for Binding Heme in B-Type Heme Proteins. Nat. Prod. Rep. 24 (3), 621–630. doi: 10.1039/B604186H 17534534

[B30] Skaar E. P. Gaspar A. H. Schneewind O. (2004). IsdG and IsdI, Heme-Degrading Enzymes in the Cytoplasm of Staphylococcus Aureus. J. Biol. Chem. 279 (1), 436–443. doi: 10.1074/jbc.M307952200 14570922

[B31] Sun X. Ge R. Zhang D. Sun H. He Q. Y. (2010). Iron-Containing Lipoprotein SiaA in SiaABC, the Primary Heme Transporter of Streptococcus Pyogenes. J. Biol. Inorg. Chem. 15 (8), 1265–1273. doi: 10.1007/s00775-010-0684-4 20607329

[B32] Tenhunen R. Marver H. S. Schmid R. (1969). Microsomal Heme Oxygenase. Characterization of the Enzyme. J. Biol. Chem. 244 (23), 6388–6394. doi: 10.1016/S0021-9258(18)63477-5 4390967

[B33] Thielges M. C. Chung J. K. Axup J. Y. Fayer M. D. (2011). Influence of Histidine Tag Attachment on Picosecond Protein Dynamics. Biochemistry. 50 (25), 5799–5805. doi: 10.1021/bi2003923 21619030 PMC3133630

[B34] Traore E. S. Li J. Chiura T. Geng J. Sachla A. J. Yoshimoto F.. (2021). Heme Binding to HupZ With a C-Terminal Tag From Group A Streptococcus. Molecules 26 (3), 1–19. doi: 10.3390/molecules26030549 PMC7865249 33494451

[B35] Tripathi S. O'Neill M. J. Wilks A. Poulos T. L. (2013). Crystal Structure of the Pseudomonas Aeruginosa Cytoplasmic Heme Binding Protein, Apo-PhuS. J. Inorg. Biochem. 128, 131–136. doi: 10.1016/j.jinorgbio.2013.07.030 23973453 PMC3843485

[B36] Tropea J. E. Cherry S. Waugh D. S. (2009). Expression and Purification of Soluble His(6)-Tagged TEV Protease. Methods Mol. Biol. 498, 297–307. doi: 10.1007/978-1-59745-196-3_19 18988033

[B37] Vagenende V. Yap M. G. Trout B. L. (2009). Mechanisms of Protein Stabilization and Prevention of Protein Aggregation by Glycerol. Biochemistry 48 (46), 11084–11096. doi: 10.1021/bi900649t 19817484

[B38] Walker M. J. Barnett T. C. McArthur J. D. Cole J. N. Gillen C. M. Henningham A.. (2014). Disease Manifestations and Pathogenic Mechanisms of Group A Streptococcus. Clin. Microbiol. Rev. 27 (2), 264–301. doi: 10.1128/CMR.00101-13 24696436 PMC3993104

[B39] Wang A. Zeng Y. Han H. Weeratunga S. Morgan B. N. Moenne-Loccoz P.. (2007). Biochemical and Structural Characterization of Pseudomonas Aeruginosa Bfd and FPR: Ferredoxin NADP+ Reductase and Not Ferredoxin is the Redox Partner of Heme Oxygenase Under Iron-Starvation Conditions. Biochemistry 46 (43), 12198–12211. doi: 10.1021/bi7013135 17915950

[B40] Watkins D. A. Johnson C. O. Colquhoun S. M. Karthikeyan G. Beaton A. Bukhman G.. (2017). Global, Regional, and National Burden of Rheumatic Heart Disease, 1990-2015. N. Engl. J. Med. 377 (8), 713–722. doi: 10.1056/NEJMoa1603693 28834488

[B41] Wilks A. Heinzl G. (2014). Heme Oxygenation and the Widening Paradigm of Heme Degradation. Arch. Biochem. Biophys. 544, 87–95. doi: 10.1016/j.abb.2013.10.013 24161941 PMC6476305

[B42] Wilks A. Ikeda-Saito M. (2014). Heme Utilization by Pathogenic Bacteria: Not All Pathways Lead to Biliverdin. Acc. Chem. Res. 47 (8), 2291–2298. doi: 10.1021/ar500028n 24873177 PMC4139177

[B43] Wissbrock A. George A. A. P. Brewitz H. H. Kuhl T. Imhof D. (2019). The Molecular Basis of Transient Heme-Protein Interactions: Analysis, Concept and Implementation. Bioscience Rep. 39, 1–11. doi: 10.1042/BSR20181940 PMC6356037 30622148

[B44] Zhang R. Zhang J. Guo G. Mao X. Tong W. Zhang Y.. (2011). Crystal Structure of Campylobacter Jejuni ChuZ: A Split-Barrel Family Heme Oxygenase With a Novel Heme-Binding Mode. Biochem. Biophys. Res. Commun. 415 (1), 82–87. doi: 10.1016/j.bbrc.2011.10.016 22020097

[B45] Zhu L. Olsen R. J. Beres S. B. Ojeda Saavedra M. Kubiak S. L. Cantu C. C.. (2020). Genome-Wide Screens Identify Group A Streptococcus Surface Proteins Promoting Female Genital Tract Colonization and Virulence. Am. J. Pathol. 190 (4), 862–873. doi: 10.1016/j.ajpath.2019.12.003 32200972 PMC7184637

[B46] Zhu W. Wilks A. Stojiljkovic I. (2000). Degradation of Heme in Gram-Negative Bacteria: The Product of the hemO Gene of Neisseriae is a Heme Oxygenase. J. Bacteriol. 182 (23), 6783–6790. doi: 10.1128/JB.182.23.6783-6790.2000 11073924 PMC111422

